# The Role of Exchange Energy in Modeling Core-Electron Binding Energies of Strongly Polar Bonds

**DOI:** 10.3390/molecules30132887

**Published:** 2025-07-07

**Authors:** Feng Wang, Delano P. Chong

**Affiliations:** 1 School of Science, Computing and Emerging Technologies, Swinburne University of Technology, Melbourne, VIC 3122, Australia; 2 Department of Chemistry, University of British Columbia, 2016 Main Mall, Vancouver, BC V6T 1Z1, Canada

**Keywords:** C1s core-electron binding energy (CEBE), density functional theory (DFT) computations, PW86x-PW91c, mPW1PW and PBE50 functionals, exchange energy, X-ray photoelectron spectra (XPS)

## Abstract

Accurate determination of carbon core-electron binding energies (C1s CEBEs) is crucial for X-ray photoelectron spectroscopy (XPS) assignments and predictive computational modeling. This study evaluates density functional theory (DFT)-based methods for calculating C1s core-electron binding energies (CEBEs), comparing three functionals—PW86x-PW91c (DFTpw), mPW1PW, and PBE50—across 68 C1s cases in small hydrocarbons and halogenated molecules (alkyl halides), using the delta self-consistent field ΔSCF (or ΔDFT) method developed by one of the authors over the past decade. The PW86x-PW91c functional achieves a root mean square deviation (RMSD) of 0.1735 eV, with improved accuracy for polar C-X bonds (X=O, F) using mPW1PW and PBE50, reducing the average absolute deviation (AAD) to ~0.132 eV. The study emphasizes the role of Hartree–Fock (HF) exchange in refining CEBE predictions and highlights the synergy between theoretical and experimental approaches. These insights lay the groundwork for machine learning (ML)-driven spectral analysis, advancing materials characterization, and catalysis through more reliable automated XPS assignments.

## 1. Introduction

Core electrons of a molecule, though typically overshadowed by their valence counterparts in chemical studies, play a foundational role in determining molecular structure and properties. Often regarded as the “deeply buried” or “frozen” portion of an atom’s electron cloud, core electrons are tightly bound to the nucleus and exhibit minimal direct involvement in chemical bonding. However, their presence is integrated into the stability of atomic and molecular frameworks. Core orbitals influence the shielding and effective nuclear charge experienced by valence electrons, thereby impacting bond lengths, bond angles, electron distributions, and molecular configurations. Neglecting the subtle contributions of core electrons can lead to incomplete or inaccurate interpretations of molecular phenomena, especially when exploring isomerization, tautomerism, or other fine structural details [1].

Core-electron binding energies (CEBEs) contain information about gas and condensed phases of matter. The 1901 Nobel Prize in Physics was awarded to Wilhelm Conrad Röntgen for his discovery of X-rays, often referred to as “magic rays”. Core-electron ionization and excitation spectroscopy, which involve removing or exciting an electron from core orbitals in a compound, require high-energy sources such as synchrotron radiation. Core ionization, for instance, is measured using techniques like X-ray photoelectron spectroscopy (XPS), whereas core excitation, on the other hand, is studied through methods like X-ray absorption spectroscopy (XAS) or near-edge X-ray absorption spectroscopy (NEXAS) [2]. XPS, also known as electron spectroscopy for chemical analysis (ESCA) [3,4,5], provides localized insight into the atomic structure outside the nucleus. This technique not only determines the chemical state of elements but also elucidates the nature of chemical bonding, offering region-specific information within a molecule. Consequently, XPS has found widespread applications, ranging from the analysis of organic molecules like pharmaceuticals to inorganic materials such as catalysts and energy storage materials. When combined with information about valence energy levels obtained from complementary spectroscopic techniques, XPS delivers a detailed, localized view of atomic interactions, chemical bonding, and oxidation states [6,7].

Molecular spectroscopy is measurable quantum mechanics [1]. Molecular spectroscopy is used to measure transitions between unique energy states, and quantum mechanics calculates the states by solving the Schrödinger equation of the compounds [8]. Advances in both experimental methodologies and theoretical models, such as density functional theory (DFT) and other quantum mechanical methods, have enabled the accurate simulation and interpretation of core-electron spectra, bridging the gap between experimental observations and molecular-level insights. As researchers continue to refine these approaches, core electrons are increasingly recognized as vital to understanding molecular structure, reactivity, and electronic behavior.

**Figure 1:**
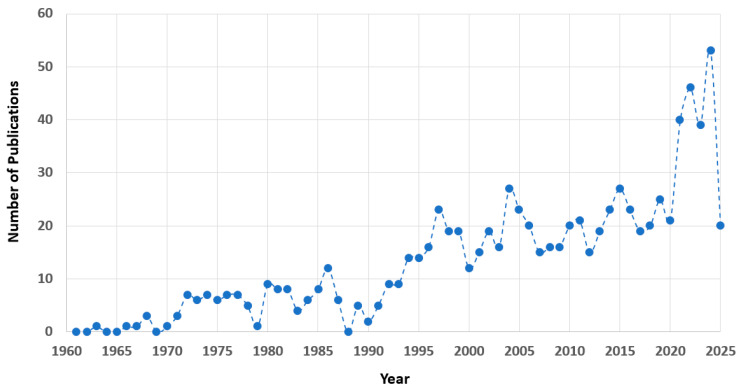
The results of the search of Scopus databases for the term “core electron binding energy of molecules”. A total of 862 documents were found as of 30th May 2025.

Gas-phase XPS has emerged as a powerful tool for probing the intrinsic properties of isolated molecules over decades [9]. The field has undergone significant advancements, fueled by the development of high-resolution synchrotron radiation sources [10]. These state-of-the-art experimental facilities provide intense, tunable X-rays capable of accessing core-electron transitions with remarkable precision. In the gas phase, subtle yet reproducible shifts in the CEBEs—known as chemical shifts—serve as a key indicator of the molecule’s chemical state. For example, the experimental binding energy (or CEBE) of the C1s electron of methane is measured at 290.703 eV [11]. Shifts from this value reflect changes in the electronic and chemical environment [3,4,5], enabling a deeper understanding of molecular structure and bonding in isolated systems. The breakthroughs in the decades of the 1990s–2010s have brought about the advent of third-generation synchrotrons. One of such synchrotron facilities that allows gas-phase XPS measurements of molecules is Elettra-Sincrotrone Trieste S.C.p.A since its commissioning in 1993, where the first beamline has operated since 1994 [10]. While it is a third-generation synchrotron facility, this Italian synchrotron light source has contributed to many breakthroughs, particularly in gas-phase spectroscopy, i.e., the GasPhase Beamline. This high-resolution XPS, which specializes in photoionization and photoelectron studies of free molecules and clusters, has allowed for high-precision measurements of CEBEs and excitation spectra, providing essential experimental benchmarks for computational approaches [10,12,13,14,15,16]. In return, computational methods also help to obtain insight into the structure of the target and identify misassignments or other phenomena [1]. For example, the study of phenylalanyl–phenylalanine (PhePhe) dipeptides using XPS highlights the necessity of integrating theory and experimental measurements to account for dehydration-induced cyclization under vacuum conditions, which produced the spectrum of a different compound, cyclo- phenylalanyl–phenylalanine [13]. Figure 1 presents the results of a Scopus scientometric search using the keywords “core electron binding energy of molecules”, yielding 862 documents. While relying on a single database may exclude studies using different terminology, the data reveal a clear trend: achieving spectroscopic accuracy in core-electron binding energy (CEBE) calculations remains a significant challenge, with synchrotron-based experimental XPS efforts driving progress in the field since the mid-1990s.

X-ray photoelectron spectroscopy (XPS) is widely employed for probing the structural and electronic properties of molecules and materials. However, assigning individual peaks to specific atomic environments remains a challenge due to the absence of comprehensive and reliable reference datasets [17]. In many cases, atoms in chemically distinct environments may exhibit nearly identical CEBEs, blurring the direct correlation between structure and spectral features. Further complications arise during peak deconvolution, which is necessary to interpret overlapping signals. This step is sensitive to experimental conditions—such as resolution and vibrational broadening (e.g., Franck–Condon effects)—and often involves assumptions regarding the number and nature of the underlying peaks [17,18]. These uncertainties can hinder confident structural interpretation from XPS data [1]. Traditionally, researchers attempt to deduce structure from a measured spectrum—a “top-down” approach. Alternatively, a “bottom-up” method involves simulating spectra from plausible structural candidates and identifying the best match with the experiment to infer the most likely molecular structure [1].

A primary challenge in computational chemistry (bottom up) lies in modeling core-electron processes. For example, in XPS, where an electron is removed from a core orbital, the quantity of interest is the energy difference between the neutral and ionized species. Traditional quantum chemical methods, optimized for valence electrons, often struggle to accurately describe the localized and high-energy nature of core orbitals. Despite advancements in experimental capabilities, the lack of robust computational tools tailored to core-level studies remains a significant bottleneck. This disconnect indicates the urgent need for theoretical frameworks capable of addressing CEBEs, chemical shifts, and electronic transitions with precision. By integrating experimental techniques with advanced quantum chemical methods, it becomes possible to achieve a more comprehensive understanding of complex chemical systems. This synergy between experiment and theory is essential for addressing pressing challenges in fields ranging from catalysis and materials science to biochemistry and environmental science. The motivation for the present study stems from this need to refine computational methodologies, bridge gaps between theory and experiment, and unlock deeper insights into the chemical and physical phenomena underlying modern molecular sciences.

Core electrons, which can be regarded as the foundation of a molecular structure akin to the unseen base of a house [19], play a pivotal role in defining the electronic framework and geometry of molecules. Despite their significance, they are frequently overlooked in computational studies, with approximations such as the frozen core approximation limiting a deeper understanding of their contributions [1]. As Ohno et al. aptly noted, many molecular properties and phenomena remain unexplained without a detailed study of core electrons [19]. Accurately calculating CEBEs for even small molecules still presents challenges. For example, even with the recently developed high accuracy of GW methods, errors of less than 0.5 eV for the first ionization energies (IPs) [20] and between 0.27–5.0 eV for four C1s CEBEs of ethyl trifluoroacetate, depending on the specific GW variant used [21], were reported—a range that remains relatively large. Notably, a recent study achieved a mean absolute error (MAE) of just 0.16 eV for absolute CEBEs using the CORE65 dataset [22]. To enable studies of larger compounds—such as pharmaceuticals, biomolecules, or high-energy storage materials—accurate training databases of CEBEs, combined with reliable theoretical or computational approaches, are essential. These can also support advanced tools such as machine learning (ML) [23,24].

In this study, we explore recent advances in applying DFT to core-electron spectroscopy, such as XPS. By examining the advantages, limitations, and accuracy of DFT methods, we aim to provide a comprehensive overview of their role in bridging experimental and theoretical studies of core-level processes. Most recent synchrotron-sourced gas-phase experiments can measure C1s CEBEs in small molecules with an accuracy of up to 0.001 eV [25], although the third decimal digits may be uncertain due to limited values for N1s, O1s, and F1s. The availability of such high-quality experimental results stimulated further study of the performance assessment of the new C1s CEBE Method C [26]. While DFT is useful, the choice of the exchange–correlation functional (V_xc_) remains critical for obtaining reliable C1s CEBE results [26]. Among the available options, the combination of the Perdew–Wang 1986 functional for exchange (PW86x) [27] and the Perdew–Wang 1991 functional for correlation (PW91c) [28] has shown promise for capturing the subtle energy shifts and localized nature of core orbitals, making it a strong candidate for detailed studies of CEBE and their transitions in molecules. By bridging the gap between theory and experiment, this study aims to validate the accuracy of the PW91c-PW86x (DFTpw) functional and demonstrate its versatility across a range of X-ray spectroscopic applications.

## 2. Core-Electron Binding Energy (CEBE) of Molecules

**Figure 2:**
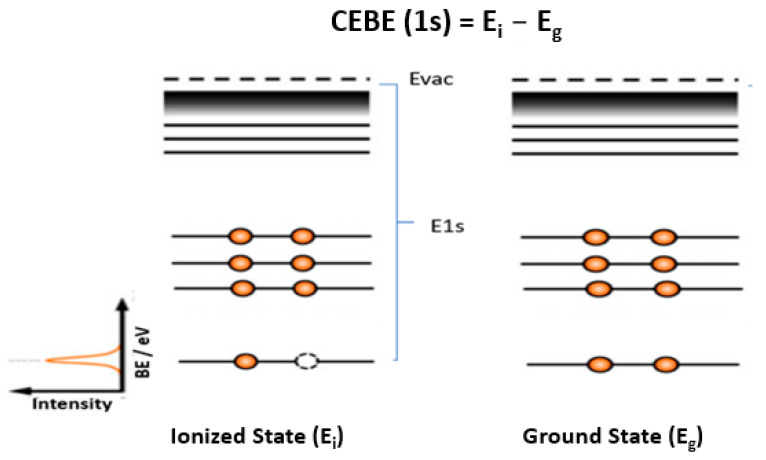
Illustration of XPS and the delta (Δ) CEBE method of a molecule. Adapted from [30] with modifications.

The CEBE of a molecule refers to the energy required to remove a core electron from an atom or a molecule. It is a fundamental quantity measured by XPS and plays a key role in characterizing the electronic structure, chemical bonding, conjugation, local electronic effects, and oxidation states of molecules and materials. Figure 2 provides an illustration of the core electron ionization process. Unlike valence ionization energies, CEBEs are rarely calculated using the Koopman theorem or meta-Koopman theorem if DFT methods are applied [29].

When a core electron is removed (see left panel of Figure 2), the remaining electrons of the cation undergo relaxation and reorganization to compensate for the missing charge. This core relaxation effect is much stronger than in valence ionization. As a result, methods such as Koopmans’ theorem (which assumes frozen orbitals) do not hold for core electrons, making such direct CEBE calculations inaccurate. Many standard DFT functionals, such as B3LYP, PBE0, and M06-2X, are optimized specifically for valence electrons, focusing on properties like bonding, reaction energies, and electronic structure, rather than CEBEs, and are often difficult to capture core relaxation and correlation accurately. Table 1 compares key challenges of obtaining CEBE and valence ionization potentials. Core electrons experience stronger electron correlation effects than valence electrons, and since core electrons are deeply bound and tightly localized, their interactions with surrounding electrons are more pronounced. The strong electron correlation requires higher-order methods like ΔSCF, GW, coupled-cluster (CCSD(T)), or perturbation theory to improve accuracy.

Methods like delta self-consistent field (ΔSCF) and transition state approaches for explicit relaxation corrections must be employed for CEBE calculations. Moreover, core electrons move at speeds close to the speed of light, especially in heavy elements (Z > 20). This leads to relativistic effects, including orbital contraction (core orbitals shrink in size) and spin–orbit coupling (splitting of energy levels due to electron spin interactions), causing standard non-relativistic methods to fail to capture these effects, leading to errors in calculated CEBEs. Therefore, relativistic corrections, such as Douglas–Kroll–Hess (DKH) [31], Zeroth-Order Regular Approximation (ZORA) [32], or relativistic Hamiltonians, are required. Unlike valence electrons, which are more delocalized, making their ionization more straightforward to approximate, core electrons are highly localized, which means that their removal creates an imbalance in local electron density. Many electronic structure methods struggle to handle this sudden localization change. As a result, localized core-hole methods (e.g., ΔSCF, Maximum Overlap Method (MOM), or ΔSCF/ECP) [33,34,35] are required. Some DFT functionals artificially delocalize charge, leading to errors in CEBE calculations. Several popular basis sets, such as standard valence basis sets and cc-pVTZ and aug-cc-pVDZ, are not optimized for core-electron description, so they are not suitable for the calculation of CEBE [26,36,37,38]. Core–valence correlation must be captured using core-augmented basis sets, such as cc-pCVnZ (core–valence correlation-consistent basis sets), uncontracted basis sets (better flexibility for core regions), and Slater-type orbitals (STOs), such as those in Amsterdam Density Function (ADF) computational software [39], which provide better core descriptions than the more popular Gaussian basis sets [26,36,37,38].

Core-hole localization and symmetry issues also become more impactful. In molecules with multiple equivalent atoms (e.g., benzene and toluene), the core-hole state may delocalize due to molecular symmetry. However, experimentally, core holes are localized on a single atom. Many computational methods tend to delocalize the core hole, artificially lowering the CEBE values. To recognize these effects, it requires localized core-hole approaches, such as ΔSCF with a localized core-hole atom basis set or the use of fragment-oriented approaches to force localization. Therefore, experimental calibration and benchmarking CEBE values often require energy referencing issues in gas-phase XPS experiments.

In addition, accurate CEBE predictions require calibration against high-accuracy experimental data.

## 3. Development of CEBE Methods

Many different approaches exist to calculate the CEBE of molecules in the gas phase. Clearly, removing an electron from a valence state increases the potential that is felt by the core electron and leads to a shift in the CEBE for the cation of binding energy (BE) or ionization potential (IP):

IP = E(cation) − E(neutral)(1)

The simplest approach, however, is the use of Koopmans’ theorem, which relates the ionization potential of an electronic state in Hartree–Fock (HF) theory with the negative energy of the relevant HF eigenstate [40]. For the closed-shell initial state, the initial state, or frozen orbital (FO), the BE is given by Koopmans’ theorem (KT) as [40,41,42]

IP_i_ (FO) = IP_i_ (KT) = −ε_i_ (2)

where ε_i_ is the orbital energy of the ionized orbital obtained from HF theory.

Other methods include delta-coupled-cluster (ΔCC) methods [43], equation-of-motion CC (EOM-CC) [44,45], GW methods [20,46,47] and beyond [20], and real-time, time-dependent (TD) DFT [48]. The GW methods and the TD-DFT methods yield mean absolute errors (MAE) of 0.24 eV and 0.27 eV, respectively. Despite the fact that in DFT, this relationship only holds for the highest occupied Kohn–Sham (KS) state [49], KS energies of core levels of a molecule are often used to estimate the chemical shift contribution to the CEBE, i.e., the displacement of the core-level before removal of the electron (also termed the initial state effect) [36,50,51,52,53].

Studies by Stener et al. [54] and Chong et al. [55,56] demonstrated the utility of the DFT transition potential (TP) for core-π* valence excitations of small molecules. Such methods are extended to larger molecules [57], and the self-consistent field (SCF) can be applied to densities referring to any state with fractional occupancy. For example, under the independent electron (one electron) assumption, a “ΔSCF” Kohn–Sham (KS) theory can be implemented using the SCF from full and zero occupancies of the core orbital SCF [57], and the IP is given by

IP = Δ(KS − SCF) + ΔE_rel_ (3)

A commonly used approach for estimating XPS CEBE within DFT is through the use of the negative KS orbital energies, as indicated in Equation (3). While conceptually straightforward, this method is limited by its neglect of electron relaxation effects (ΔE_rel_) and can be particularly problematic for molecules with high symmetry, such as benzene [58]. In such systems, the KS orbitals corresponding to symmetry-equivalent atomic sites may form delocalized combinations of atomic core orbitals. When these atoms are spatially proximate, their core orbitals can interact significantly, resulting in the formation of bonding and antibonding combinations, thereby altering the computed energy levels—lowering the energy of the bonding state and raising that of the antibonding one.

Despite this, KS SCF calculations often produce stable results that align well with previous SCF or MCSCF findings [59], or in some cases, even offer improved agreement [57]. Still, IPs derived from the core 1s_1_/_2_ orbitals under transition potential approximations show noticeable dependence on the chosen functional and may require systematic corrections. Interestingly, the 1s_1_/_2_ transition potential not only accounts for relaxation when compared to Slater-type exchange (STEX) data but also mitigates some of the deficiencies seen in SCF methods related to π*-orbital screening. In some instances, it can approach the accuracy expected from more computationally intensive electron correlation treatments [57].

## 4. Overview of DFT Methods for Accurate CEBEs

The longstanding interest of Chong et al. in calculating CEBEs for small molecules dates back to 1983 [60], when Chong et al. developed an ab initio transition operator method (TOM) [61] to account for relaxation effects, combined with Rayleigh–Schrödinger perturbation theory (RSPT) to incorporate correlation effects, which is referred to as [60]

Method A (TOM + RSPT)(4)

Method A in Equation (4) was initially applied to calculate vertical IP for core orbitals in small molecules such as HF, H_2_O, and CO [60,62]. The results were further refined by employing third-order Rayleigh–Schrödinger perturbation theory, leading to significantly improved accuracy. At the time, the method demonstrated remarkable reliability, achieving an average absolute deviation (AAD) from experimental CEBEs of 0.4 eV [60]—a notable achievement over forty years ago. However, due to the computational limitations in the 1980s–1990s, these intensive calculations were largely restricted to small molecules, making it challenging to extend the methodology to medium-sized systems.

The natural remedy was to turn to density functional theory (DFT). When using the DFT methods, one faces the problem of the correct choice of the exchange–correlation (V_xc_) functional to describe the quantities of interest [63], as there is no clear improvement in the reliability and accuracy of describing the electron density distribution while climbing Jacob’s Ladder. The next development in the period of the mid-1990s [36,52,53,55,56,64,65] involved a model known as the unrestricted generalized transition state (uGTS) [36]:

Method B = uGTS(DFT − V_xc_)/scaled pVTZ (5)

In the uGTS method, unrestricted (u) means that the calculation allows different spatial orbitals for alpha (spin-up) and beta (spin-down) electrons, which can better describe open-shell or partially ionized systems where spin symmetry is broken. The GTS refers to a method where the core orbital of interest is assigned a fractional occupation (often half an electron removed, i.e., 0.5 occupation), instead of being fully occupied (1.0) or fully ionized (0.0). This fractional occupation mimics the transition state between the ground and core-ionized states, capturing some relaxation effects without a full ionization calculation [36]. As a result, uGTS approximates the core ionization energy by calculating the energy of a system with a half-occupied core orbital under an unrestricted spin framework. This approach balances accuracy and computational cost by incorporating orbital relaxation effects more effectively than simply using orbital energies, but with less computational effort than full core-ionized state calculations.

Here, the DFT-V_xc_ functional is usually the Becke88x-Perdew86c functional for C1s CEBE of molecules. More details of the development have been summarized in a review article in 2002 [66]. For over 200 molecular cases, including some medium-sized molecules, the AAD was reduced significantly to 0.2 eV. However, the good performance of Method B was a result of a fortuitous cancelation of the uGTS model error by the functional error. Despite this, Method B continued to be used until 1999, when an improved DFT approach, Method C [26], was developed:

Method C = ∆(DFTpw) + C_rel_ (6)

This method was developed based on comparison with experimental values, conveniently compiled by Bakke et al. [67] and updated by Jolly et al. [68]. Here, C_rel_ is a scalar relativistic effect for C, N, O, and F atoms, and is included as a *post hoc* correction developed by Chong et al. [26,52,69] for C, N, O, and F. It led to the following formula:

(7) $$C_{\mathrm{rel}}=A\;I_{nr}^{B}$$

where the correction C_rel_ (in eV) is found with constants A = 2.198 × 10^−7^ eV, B = 2.178, and the calculated non-relativistic core IP I_nr_ (in eV) [26,52,69]. The relativistic corrections for carbon (C1s), nitrogen (N1s), oxygen (O1s), and fluorine (F1s) are 0.05 eV, 0.1 eV, 0.2 eV, and 0.35 eV, respectively [26,52,69]. Other studies found that the relativistic corrections for carbon (C1s) can be as high as 0.12–0.13 eV [47,70].

**Figure 3:**
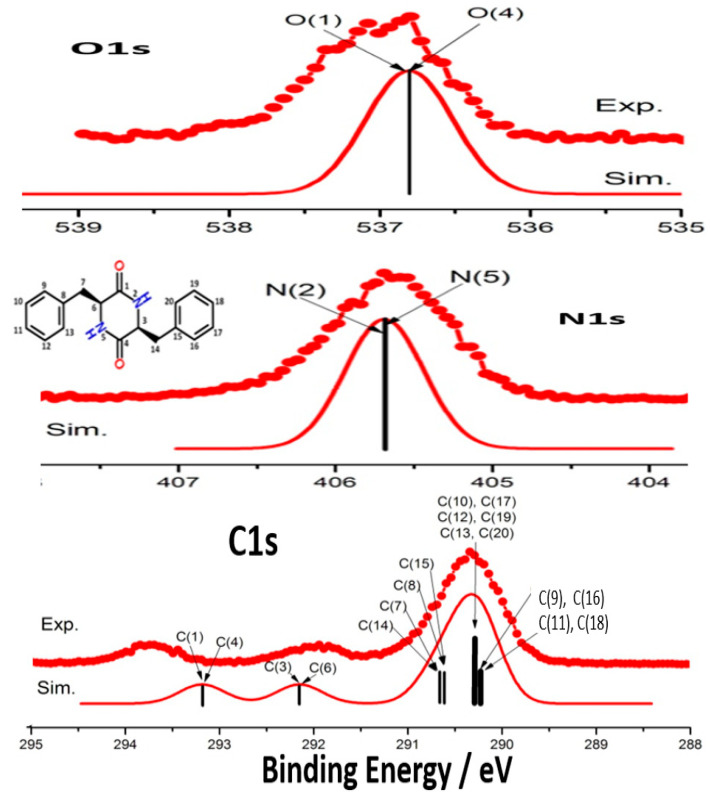
A comparison of the measured and calculated C1s, N1s, and O1s XPS spectra of the cyclo-dipeptide, c(phenylalanyl–phenylalanine). The spectrum was replot based on the results of Ref. [13].

As a result, Method C in Equations (6) and (7) has been applied to many molecules since the beginning of this century (2002), providing essential theoretical support to XPS experiments and their assignment [1,26,52,69]. The encouraging results were also summarized in a recent paper [71]. The calculation of CEBEs for molecules containing C1s, N1s, O1s, and F1s has been well documented [72]. With the development of synchrotron-sourced XPS in the gas phase in the early 2000s, accurate CEBE calculations became important in the interpretation of the measured C1s, N1s, and O1s XPS spectra of medium-sized molecules such as amino acids [73,74,75,76,77,78], DNA bases [10,16,76,79], dipeptides [13,80], and other biologically important molecules [81,82], drugs [83,84,85], drug candidates [86,87] and energy storage materials [88,89,90] with high accuracy. Figure 3 presents the comparison of C1s, N1s, and O1s XPS measured in the gas phase and the calculated CEBE of cyclo-phenylalanyl–phenylalanine using Method C in Equation (6) [13].

Molecules containing strong polar bonds such as C-X (X=O and F) result in significant charge separation, which poses a challenge for purely DFT-based methods that tend to underestimate exchange effects. The mPW1PW functional [91] was developed to enhance the accuracy of both covalent and noncovalent interactions by modifying the exchange functional [92,93] originally introduced by Perdew and Wang (PW91) [28]. The need for mixing the exact Hartree–Fock (HF) exchange energy with density functional approximations was later justified by Perdew and others [94,95], providing a theoretical foundation for hybrid functionals. To optimize its performance, the inclusion of different ratios of the HF exchange energy at 0.20, 0.30, 0.40, and 0.50 was tested for the CEBE calculations in small molecules, such as trifluoromethylacetate [96]. These studies revealed that an HF exchange contribution of approximately 50% provided an excellent agreement with experimental CEBE for molecules with large polar bonds such as CO_2_ and CF_4_, which otherwise exhibited significant C1s CEBE errors [96,97]. A key advantage of mPW1PW is that it expands the applicability of DFT calculations while preserving the asymptotic and scaling properties of the original functional, without introducing additional adjustable parameters [92,93]. It incorporates an adiabatic connection method, where the ratio of exact HF exchange to DFT exchange is determined from purely theoretical principles, eliminating the need for empirical tuning. This theoretical consistency makes mPW1PW particularly effective for modeling highly polar bonds in small molecules [96]. By incorporating a balanced amount of HF exchange, the mPW1PW functional improves the description of exchange interactions in these highly polar systems, leading to more accurate predictions of valence ionization energies [96]. Furthermore, its ability to maintain correct scaling conditions and accurately describe both localized and delocalized electron interactions enhances its reliability in handling small molecules with strong C-X polar bonds.

The inclusion of α = 50% HF exchange energy has significantly enhanced the performance of the mPW1PW functional [96]. In this study, we introduce the PBE50 functional [98] to calculate core electrons of molecules, which has been previously employed in many-body perturbation theory in the G_0_W_0_ approximation to study valence ionization energies of molecules [99]. The PBE50 functional is an extension of PBE0 [94,100], increasing the HF exchange from α = 25% (PBE0) to α = 50% (PBE50). This adjustment aims to improve accuracy in systems where exchange effects play a critical role. The modification follows the reasoning proposed by Perdew [98], who suggested that increasing the exact exchange contribution can enhance the description of specific properties [94,98], such as barrier heights [101] and IPs, by mitigating self-interaction errors commonly present in density functional approximations (DFAs) [98]. For C1s CEBE calculations, the higher proportion of HF exchange in PBE50 leads to more accurate predictions, particularly in small alkane halides (C-X systems, where X=(O), F, Cl, Br). Compared to the PW86x-PW91c (DFTpw) functional and other generalized gradient approximation (GGA) functionals, PBE50 offers a significant advantage in capturing exchange interactions in highly polar bonds, where electron density distortion is more pronounced. It is also discovered that using the PBEh (α = 45%) functional using the GWA method, the BEs can be simulated within an error of 0.3 eV with respect to the experimental BEs from as large as 1.2 eV for C1s, N1s, O1s, and F1s [20].

## 5. Computational Details

The computational framework for the present study combines advanced DFT functionals with carefully chosen basis sets and software to ensure the reliability of the results. In earlier studies, Gaussian basis sets [26,37] such as the correlation-consistent polarized core–valence triple-zeta (cc-pCVTZ) in the Gaussian computational chemistry package [102]. However, contracted basis sets like the cc-pVnZ series, which are single-zeta in the core region and n-zeta in the valence region, lack the flexibility necessary for describing core–valence interactions. To address this limitation, uncontracted Gaussian basis sets were utilized in some cases. For most of our studies, including the present work, we employed the Amsterdam Density Function (ADF) program [39], leveraging its efficient Slater-type orbital (STO) basis sets. Specifically, the et-pVQZ basis set [103], which is double-zeta in the core region and quadruple-zeta in the valence region, was selected to ensure an accurate representation of the electronic structure. More importantly, this Slater-type basis set is more flexible in the core region than the aug-cc-pVTZ or cc-pVQZ Gaussian-type orbital (GTO) basis sets used in Gaussian-based programs for valence space. For a more comprehensive discussion of the role of basis sets in the calculation of CEBEs of small molecules, please refer to a recent article by Delgado and Matthews [45].

The use of CCSD(T)-optimized geometries in ΔDFT calculations of C1s CEBEs is motivated by their ability to provide highly accurate and physically realistic descriptions of local electronic environments. Since C1s CEBEs are extremely sensitive to the immediate chemical surroundings of the carbon atom, including bond lengths and angles, even minor geometric inaccuracies can result in significant deviations, often by tenths of an eV. CCSD(T). This is widely regarded as the “gold standard” for small molecules (typically fewer than 10 atoms), delivering highly reliable equilibrium geometries and accurate electron densities near the nucleus, which are essential for reproducing core-level shifts. These geometries serve as an excellent reference for benchmarking DFT-level CEBE predictions and ensure internal consistency between the neutral and core-ionized states in ΔDFT. Furthermore, when geometry is optimized at lower levels of theory (such as B3LYP or HF), inaccuracies in bond metrics may lead to under- or overestimation of the core orbital energies, reducing the reliability of the calculated CEBEs. In contrast, CCSD(T)-level structures improve the accuracy of the total energies involved in ΔDFT and enhance alignment with experimental XPS measurements, offering a well-balanced approach between computational cost and spectroscopic fidelity.

The details used in this study can be summarized as follows. Geometry optimizations were performed primarily at the molecular orbital theory coupled cluster CCSD(T) level [104,105,106], with some cases using the B3LYP hybrid functional [107,108], cc-pVTZ basis set, and Slater-type basis set in the ADF program [39]. Harmonic vibrational frequencies were calculated to characterize the stationary points located on the potential energy surface. Self-consistent field (SCF) convergence was achieved to a threshold of 10^−6^ E_h_, and the core–valence relativistic corrections (C_rel_) were included to account for relativistic effects [26,52].

## 6. Materials and Methods

The target molecules for C1s CEBE calculations in this method are small- to medium-sized molecules with less than 20 atoms, containing carbon, hydrogen, nitrogen, oxygen, and halogen. The same method was employed to calculate C1s, N1s, and O1s CEBEs for larger biomolecules to satisfy the accuracy of synchrotron experiments [80]. In this article, we demonstrate on C1s only for achieving advanced accuracy. The ΔSCF method in Equation (3), while relatively simple and general, presents technical challenges when localizing and optimizing the core-hole state, particularly in molecules with multiple carbon atoms [109]. Accurately localizing the core hole is crucial for achieving reliable computational results but is inherently complex in such systems. To address these challenges, fragment-oriented approaches have been developed [38], wherein the core hole is optimized independently of the rest of the molecule. This strategy provides an alternative pathway for improving accuracy by isolating the effects of core ionization and reducing computational ambiguities [38].

Building upon these advancements, such techniques have been integrated with the proposed refinement procedure to enhance the precision of core-hole localization [69]. Specifically, a localized core-hole atom basis set is assigned to the core hole of the target atom, offering a practical and systematic solution [69]. However, this approach may introduce minor numerical errors in energy calculations. To address these discrepancies, Chong proposed a refinement method that quantifies and corrects these errors [110]. For instance, in the case of trans-1,3-pentadiene (C_5_H_10_), a correction of 0.057 eV is calculated by comparing results obtained with and without the localized core-hole atom basis set. This refinement significantly improves the agreement of calculated CEBEs with experimental data, as shown in Table 2. By combining the ΔDFT approach with fragment-oriented techniques and our refinement procedure, the challenges of core-hole localization are effectively mitigated. These methodologies collectively provide robust tools for accurately modeling core-electron processes, facilitating precise predictions of CEBEs [110]. Further analysis of trans-1,3-pentadiene demonstrates the efficacy of this approach and indicates its potential for broader applications in computational studies of core-electron spectroscopy.

As can be seen in Table 2, in trans-1,3-pentadiene (C_5_H_8_), the five carbon atoms exhibit distinct C1s CEBEs, reflecting subtle differences in their local chemical environments despite the molecule’s apparent symmetry. The terminal methylene carbon (C1, =CH_2_) shows the lowest CEBE at 289.800 eV (Method-II), due to its electron-rich, less substituted environment, which leads to greater electron density and reduced deshielding. The internal sp^2^-hybridized carbons involved in the conjugated diene system—C2 (290.571 eV), C3 (290.333 eV), and C4 (290.153 eV)—exhibit progressively lower CEBEs, consistent with π-electron delocalization and lower local electron density. The other terminal methyl carbon (C5) has the highest CEBE at 290.784 eV, likely due to its greater deshielding from conjugation and more attached hydrogens. These trends illustrate how CEBEs sensitively reflect electronic structure and bonding environments. Overall, the calculated values show excellent agreement with experimental data, with a root mean square deviation (RMSD) of just 0.1044 eV, confirming the accuracy of the computational method employed.

For molecules with two or more equivalent carbon atoms, however, this refinement cannot be applied due to the necessity of using a localized rather than a delocalized core hole. This limitation highlights a broader trend observed in DFT calculations: Integer occupations with incorrect symmetry are often favored over fractional occupations with correct symmetry. This phenomenon extends beyond core ionizations to valence ionizations as well, as previously noted in the literature [96,111]. The correction is determined by comparing calculations performed with and without the localized core-hole atom basis set. For trans-1,3-pentadiene, this correction amounts to 0.057 eV, which brings the corrected CEBEs closer to experimental values compared to uncorrected values derived from core-hole cations. In simpler cases, such as CH_3_CH_2_F and HC(CH_3_)_3_, the correction is negligible, around 0.001 eV, likely attributable to round-off errors. Overall, while the refinement introduced using localized core-hole atoms as the starting fragment provides only minor adjustments, it represents an important step toward improving the accuracy of CEBE predictions, particularly for molecules with non-equivalent carbon environments. These corrections are small but meaningful refinements, emphasizing the nuanced interplay between computational methods and molecular symmetry in achieving reliable results.

Several alternative approaches have been introduced to improve the reliability of core-hole localization and stabilization in computational simulations [109]. A commonly used technique starts from ground-state localized molecular orbitals (MOs) and selectively removes an electron from the target core orbital to model the ionized state. To aid convergence, Besley and co-workers developed the Maximum Overlap Method (MOM) [33,34,35], which determines orbital occupancies by maximizing the overlap between orbitals from consecutive iterations, rather than relying on the standard Aufbau principle [109]. This technique reduces the likelihood of variational collapse and is especially valuable for accessing core-excited states in complex or larger molecular systems. Another strategy to prevent undesired delocalization of the core hole involves employing a hybrid basis set scheme: The atom undergoing ionization is treated using an all-electron basis set, while the remaining atoms are represented with effective core potentials (ECPs). This ΔSCF/ECP method [112] effectively confines the core-hole to the target atom by design.

To further reduce the errors of the calculations for comparison with experiments, the study on the seventeen cases was carried out before the reliable synchrotron results were available and was the basis for the use of the PW86xPW91c functional to train Method C for accurate CEBEs, which can be considered as the machine learning (ML) training database for XPS. The C1s CEBEs of small molecules using Method C and DFTpw are summarized in Table 3 for a total of 68 carbon sites (C1s CEBEs), which are arranged in ascending order of the CEBEs observed in synchrotron radiation experiments. The carbon atom positions of all molecules are clearly marked in the structures in the table, except for some mono-substituted phenyl derivatives, such as toluene and fluorobenzene, which are present later (in Figure 7). An immediate examination of Table 3 shows that Method C (Equation (6)) performs very well except for carbon atoms with high positive Mulliken charges Q on the target carbons, which are also reported in Table 3. The AADs of these C1s CEBEs presented in Table 3 from the experiment are, respectively, 0.113 eV, 0.073 eV, and 0.377 eV, and for all 68 carbon sites, the top 59 cases and the last 9 cases with C-X (=O, -F) bonds are presented (highlighted in Table 3). Apparently, the last nine cases attract larger than expected errors. The root mean square deviation (RMSD) of the calculated and the measured CEBEs in Table 3 is 0.1735 eV for all 68 carbon sites. The CEBEs calculated using the present method (∆DFTpw/et-pVQZ//CCSD(T)/cc-pVTZ + C_rel_) exhibit an excellent agreement with experiments, comparatively more accurate than the recent (GW̥Γ) method [20] of several molecules such as CH_4_, C_2_H_6_, CO, CO_2_, and CF_4_. For example, the GWΓ method almost always underestimated the CEBEs for all core CEBEs of the molecules in the study; the discrepancies between the calculated and experimental C1s CEBEs of these molecules ranged from −1.02 eV for C_2_H_6_ to −2.98 eV for CH_4_. In the present study, the errors for C_2_H_6_ are 0.25 eV and −0.04 eV for CH_4_. However, the largest error in the present study is −0.61 eV for CF_4_.

In Table 3, the number of hydrogen atoms and the hybridization affect the C1s CEBE values. For example, carbons with more hydrogens, such as methyl carbons in CH(**C**H_3_)_3_ (290.39 eV), have lower C1s CEBEs compared to carbons with fewer hydrogens (e.g., **C**H(CH_3_)_3_, 290.63 eV). This occurs because carbons with more hydrogen atoms retain higher electron density, reducing their BE, whereas carbons with fewer hydrogens (but more alkyl groups) experience greater electron withdrawal, increasing their CEBE. Carbon atoms with sp^3^ hybridization exhibit more C1s CEBE than carbons with sp^2^ hybridization for the same reasons. For example, **C**H_2_=CHCH_3_ results in 290.23 eV, whereas CH_2_=CH**C**H_3_ results in 290.86 eV, which is slightly larger. Halogen substitution further influences the C1s CEBE values. For example, in **C**H_3_CH_2_F, the C1s CEBE is 291.13 eV, which is higher than 290.71 eV in CH_3_CH_3_. This increase is due to fluorine’s strong electronegativity and inductive electron-withdrawing effect, which reduces electron shielding around the carbon core electrons, making them more tightly bound and requiring higher energy for ionization.

**Figure 4:**
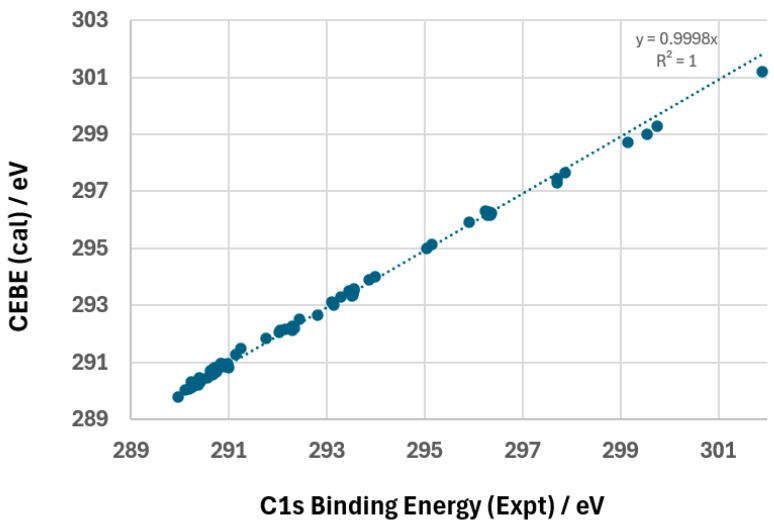
Comparison of all calculated C1s CEBE for molecules in Table 3 with respect to their XPS measurements. The correlation coefficient R^2^ of nearly 1 indicates that the calculated and measured CEBEs of the molecules agree well.

The calculated and measured CEBEs of the molecules in the database in Table 3 agree well (second last column), particularly in the top 59 cases, except for the bottom 9 cases (highlighted in the table). The Cartesian coordinates of all optimized structures are provided in Table S1 of the Supplementary Materials. Figure 4 compares the correlation between the calculated and measured values of all 68 C1s CEBEs of the small molecules in Table 3. It demonstrates an exceptional level of accuracy, with the linear regression correlation coefficient R^2^ value of ~1.000, indicating that the computed CEBEs agree well with the measured ones. Furthermore, the fitted equation of y = 0.9998x shows that the calculated values deviate negligibly from the experimental data, with a slope nearly equal to 1. This result validates the reliability of the computational method employed, confirming that the DFTpw functional combined with the chosen basis set provides highly accurate predictions of CEBEs for these molecules.

The performance of the DFTpw functional in the calculation of C1s CEBE is summarized in Table 3 and Figure 4, showing an AAD of 0.14 eV for the first 30 cases, demonstrating its high accuracy. A detailed analysis of the deviations between the DFT-calculated and experimental C1s CEBE (Δ(DFT − Expt)) reveals variations in accuracy across different molecular systems. While most of the deviations remain within 0.1 eV, as shown in the box region of Figure S1 in the Supplementary Materials, approximately 24 C1s CEBEs exhibit deviations greater than 0.1 eV, with eight cases exceeding 0.2 eV. In most cases, the calculations tend to underestimate the C1s CEBEs (ΔIP < 0), particularly for molecules containing unsaturated C=C bonds and strongly polarized C-X bonds (where X is a highly electronegative atom). For instance, the C1s energy of **C**H_2_=CH–CH=CH–CH_3_ shows a deviation of −0.17 eV, indicating a slight underestimation. The largest deviation observed in this study is for **C**F_4_, with a discrepancy as large as −0.71 eV, likely due to the significant electron-withdrawing effect of fluorine, which is challenging to capture accurately within the chosen DFT functional. These findings highlight the need for further refinement in computational approaches, particularly for molecules with strong electron-withdrawing substituents or extensive delocalization effects.

**Figure 5:**
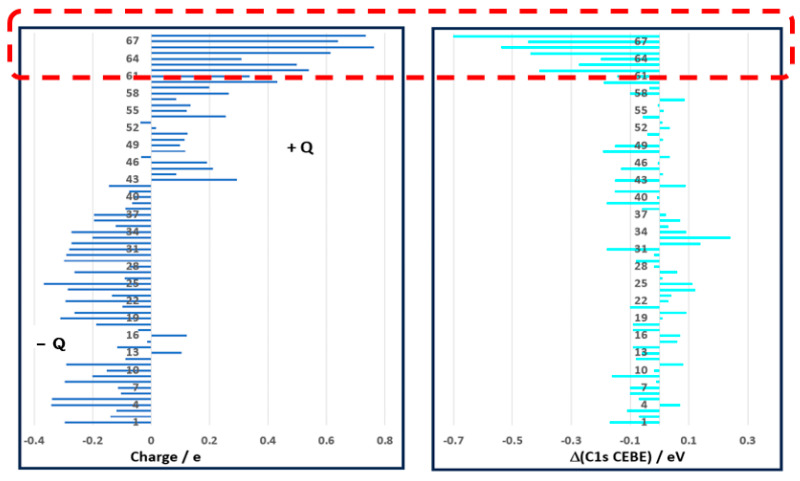
Tornado plot for the correlation between the Mulliken charge (Q) on carbons and the access Δ(C1s CEBE) on the target carbon atom of the molecules. The excess individual C1s CEBEs of the molecules with respect to their corresponding measured C1s energies. Most of the excess errors are within the CEBE ≤ ±0.1 eV box (RMSD = 0.1735 eV).

As indicated by Domagala et al. [63] that the DFT method undoubtedly has the greatest problems with a reliable description of the electron density distribution in multiple strongly polar bonds, such as C=O, and bonds associated with large electron charge delocalization. Our analysis suggests that the accuracy of the target C1s CEBEs is primarily influenced by the polarity of the individual C-X bonds, as reflected in the Mulliken charge Q on the carbon atom. Figure 5 correlates the calculated Mulliken charge (Q) on carbons (left) and the access Δ(C1s CEBE) (right) on the target carbon atom of the molecules. A key observation is that when the target carbon atom carries a large positive Mulliken charge (e.g., greater than Q > 0.3 e), the deviations between DFT-calculated and experimental C1s CEBEs (Δ(DFT − Expt)) tend to be more pronounced. This trend is particularly evident for the bottom nine C1s CEBEs with the largest discrepancies, as indicated in the dashed box in Figure 5. Here, it is the charge, not the dipole moment of the molecules, that matters as molecular symmetry may cancel out individual bond polarities, leading to a zero dipole moment in highly symmetric molecules, such as CO_2_. The charge on C is as large as 0.5394 e, but the linear symmetry of CO_2_ results in a non-dipole molecule of CO_2_. Similarly, the C-F bonds of CF_4_ are strong polar bonds, and the charge accumulated on the C atom is as large as 0.7352 eV, but due to the tetrahedral symmetry, the dipole moment of CF_4_ is zero. As indicated in Figure 5, there is a general negative correlation between the charge on the target carbon atom (Q > 0) and the Δ(C1s CEBE) < 0. That is, the more positive charge on a carbon atom, the larger the error of the calculated C1s CEBE with respect to the measured one. For example, the largest charge on the target carbon atom is Q = +0.7352 eV of CF_4_, and the Δ(C1s CEBE (DFT − Expt)) is as large as −0.71 eV, which is also the largest (negative) C1s CEBE discrepancy in Table 3.

**Figure 6:**
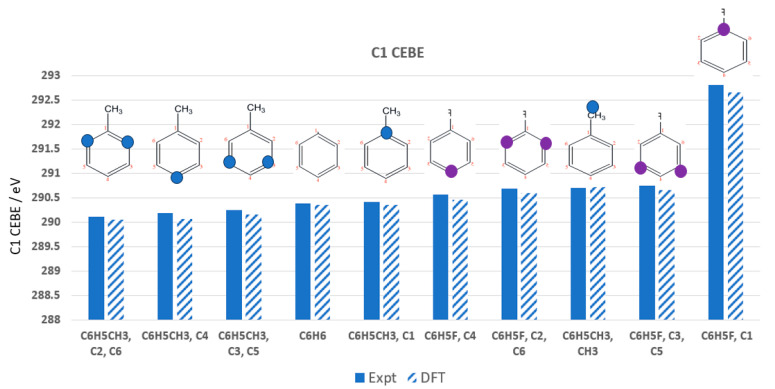
Comparison of C1s CEBEs of the mono-substituted benzenes (toluene and florobenzene) with respect to their corresponding measured C1s energies (eV) (RMSD = 0.0897 eV).

Figure 6 compares C1s CEBEs of the small hydrocarbons with respect to their corresponding measured C1s energies (RMSD = 0.0946 eV). The calculations using the DFTpw functional demonstrate remarkable accuracy for hydrocarbons, particularly saturated hydrocarbons. The average absolute deviation between calculated and experimental values is only 0.07 eV, indicating that the functional effectively captures the core-electron interactions in these systems. For saturated hydrocarbons, such as alkanes, the core-electron environment is relatively localized and experiences minimal π-delocalization effects or strong polarization interactions. This results in a well-defined CEBE that is less sensitive to electron correlation and relaxation effects, making it easier to model accurately using DFT. Furthermore, since alkanes have only σ bonding (sp^3^ hybridization), the electronic relaxation upon core ionization follows a predictable pattern, further contributing to the reliability of the computed CEBEs. On the other hand, unsaturated hydrocarbons, such as alkenes and alkynes, introduce π-electron delocalization, which affects the relaxation energy after core ionization and can introduce larger deviations in computed CEBEs. Despite this, the DFTpw functional still performs well across different hydrocarbon classes, making it a robust and reliable choice for modeling C1s CEBEs, especially when compared to high-resolution synchrotron radiation experiments.

The C1s CEBEs of molecules are influenced by their composition, structure, bonding, local atomic environment, and bond polarity in C–F bonds. In toluene (C_6_H_5_-CH_3_), the C1s CEBEs follow this order: C2, C6 (ortho) < C4 (para) < C3, C5 (meta) < C1 < CH_3_ (see Figure 6 for aromatic carbon labeling). However, when the methyl group is replaced by fluorine in fluorobenzene (C_6_H_5_-F), the order of para- and ortho-carbons is reversed: C4 (para) < C2, C6 (ortho) < C3, C5 (meta) < C1. This reversal occurs due to the electron-withdrawing effect of fluorine, which alters the electronic distribution within the benzene ring. Other C1s CEBE trends are also observed. For example, the C1s CEBEs of saturated (sp^3^) carbons are lower than those of unsaturated (sp^2^) carbons. This is because unsaturated carbons exhibit increased s-character and electron-withdrawing effects, leading to higher C1s CEBEs.

The methyl group (-CH_3_) in toluene donates electron density, reducing the CEBEs of the ortho- and para-carbons in toluene, whereas the fluorine (-F) group withdraws electron density, increasing CEBEs overall but favoring the para-carbon due to resonance effects. Bond polarity and substituent effects dictate C1s CEBE ordering, and substitution with an electronegative atom like fluorine significantly alters the electronic structure of the benzene ring. As a result, small changes in molecular structure, such as substituent effects, can lead to measurable shifts in core-electron binding energies, which are crucial for understanding XPS results.

The discrepancies between the calculated and measured C1s CEBEs of small halogen-substituted hydrocarbon molecules are, in general, small except for halogenated methane (CH_a_X_b_). Figure S3 presents the Δ(DFT − Expt) values for all halide molecules listed in Table 3. The molecules with the largest deviations (Δ > 0.2 eV) predominantly fall into two categories: (a) Unsaturated molecules (e.g., acetylene (CH≡CH) and carbon dioxide (O=C=O)): These systems involve delocalized electronic structures and strong π interactions, which may introduce additional relaxation or correlation effects not fully captured by standard DFT functionals. (b) Halogenated methane with strong, polar C-X bonds (e.g., X=F): The presence of highly electronegative halogens significantly alters electron density distribution, leading to complex relaxation effects and highly accumulated electron density, which possibly deviate in computed C1s CEBEs. It suggests that while the DFTpw functional generally provides highly accurate C1s CEBEs for many small molecules, certain electronic environments—particularly those involving π delocalization and strong polarization effects—may require further methodological refinements or corrections, such as larger exchange energy treatments or relativistic effects for heavier elements.

The DFTpw functional remains highly reliable, except for molecules with strongly polar C-X bonds, where significant deviations of C1s CEBE are as large as RMSD = 0.2143 eV. A further examination reveals that the last nine cases of halogenated methanes all contain at least one F atom, except for CO_2,_ with a large electronegativity. This is also discovered in the most recent GWT method, which underestimates the experimentally determined absolute BEs to be as high as 5.8 eV for F1s for small-sized molecules [20]. And the F1s BE has the largest error calculated using the same method. To address this limitation, the PBE50 and mPW1PW functionals with large exchange energy are employed to replace the DFTpw functional in Method C (Equation (6)). Table 4 compares C1s CEBEs of the last nine molecules with strongly polar C-X bonds, using both the DFTpw and PBE50 functionals. The PBE50 functional significantly improves the accuracy of Method C for these molecules, where large charge separation and polarization effects tend to introduce errors.

**Figure 7:**
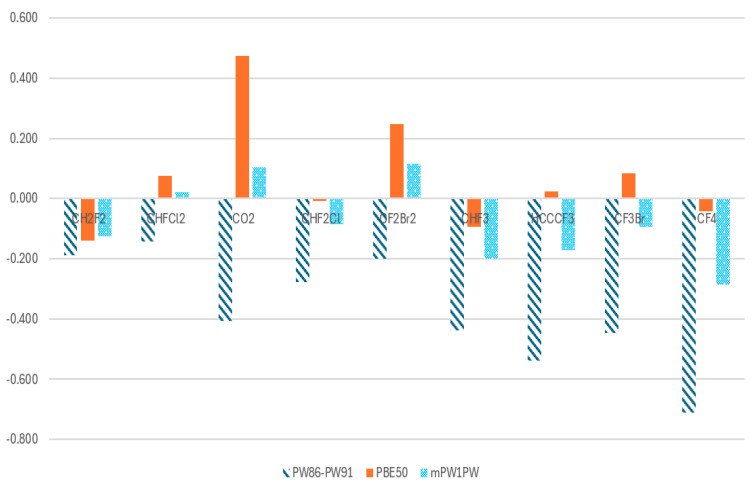
Comparison of C1s CEBEs for the last nine cases of molecules with strongly polar C-X bonds using the DFTpw functional and the new PBE50 and mPW1PW functionals.

Figure 7 presents a comparison of the C1s CEBEs for the last nine cases of molecules with strongly polar C-F(O) bonds. Incorporating a higher proportion of Hartree–Fock (HF) exchange, the PBE50 functional significantly improves accuracy, reducing the AAD from 0.38 eV (Table 3) to 0.13 eV (Table 4)—a nearly threefold improvement. This highlights the crucial role of exchange treatment in DFT functionals when addressing highly polar bonds in XPS calculations. As shown in Figure 7, both the PBE50 and mPW1PW functionals effectively reduce errors in C1s CEBE calculations using Method C. For most cases, PBE50 performs well, keeping Δ(DFT − Expt) below 0.20 eV, except for two notable exceptions: CO_2_ and CF_2_Br_2_. The Δ(DFT − Expt) for CO_2_ is 0.475 eV, even larger than the deviation obtained with DFTpw (−0.407 eV). Similarly, CF_2_Br_2_ shows an error of 0.247 eV, again exceeding the value from DFTpw. These results suggest that 50% HF exchange in PBE50 does not fully capture the electronic effects in these particular systems. In contrast, the mPW1PW functional exhibits a different performance trend, reducing the Δ(DFT − Expt) for CO_2_ and CF_2_Br_2_ to 0.104 eV and 0.115 eV, respectively. However, this functional introduces larger errors for other molecules, with deviations of −0.286 eV for CF_4_ and −0.201 eV for CHF_3_. This indicates that while mPW1PW improves accuracy for some systems, it still struggles with strongly polar fluorinated molecules, underscoring the trade-offs in functional selection for CEBE calculations.

For molecules with highly polar C-F bonds (and O=C=O), standard DFT functionals such as DFTpw tend to underestimate the C1s CEBE due to overdelocalization of electrons and poor treatment of exchange effects. Increasing the HF exchange energy (e.g., in PBE50 or mPW1PW) improves the accuracy of CEBE predictions by better describing electron localization, reducing self-interaction errors, and enhancing the treatment of exchange–correlation effects in polar bonds. This explains why hybrid functionals with increased HF exchange energy perform significantly better for molecules like CO_2_ and CF_4_, yielding CEBEs much closer to experimental values.

## 7. Conclusions

This study provides a concise overview of the evolution and development of accurate C1s core-electron binding energy (CEBE) calculations for small molecules using DFT-based methods developed by Chong et al. over the past decades. Using three density functionals—the standard PW86x-PW91c (DFTpw) and mPW1PW and PBE50 enhanced by the HF exchange energy (V_xc_ = 50%)—the C1s CEBEs of 68 carbon sites of a set of small molecules were calculated with high accuracy, achieving a total RMSD of 0.1735 eV. The accuracy further improved when the new DFT functionals enhanced with more HF exchange energy, mPW1PW and PBE50, were applied to the last nine molecules, which contain strongly polar C-X bonds, such as CO_2_ and CF_4_. Each of these DFT functionals offers distinct advantages: PW86x-PW91c (DFTpw) provides a reliable baseline accuracy, mPW1PW effectively accounts for large C-X bond polarities, and PBE50 enhances accuracy by incorporating a higher fraction of HF exchange. The average absolute deviation (AAD) of C1s CEBEs computed using Method C with the PW86x-PW91c functional was 0.113 eV across all 68 molecules. Notably, when excluding the last nine cases with strong C-X bonds, the AAD improved to 0.073 eV for the first fifty-nine molecules. However, for the last nine cases with C-F (and CO_2_), the AAD of 0.372 eV using the PW86x-PW91c functional was significantly reduced to 0.132 eV and 0.133 eV using the modified PBE50 and mPW1PW functionals, respectively. The consistency of these results across a wide range of molecular systems highlights the reliability of DFT for XPS assignments, providing a valuable reference for both theoretical and experimental studies.

Beyond accurate spectral predictions, the integration of theoretical methods with experimental studies not only validates experimental observations but also unveils the fundamental principles governing core-electron behavior. This synergy deepens our understanding of molecular structure, bonding, and electronic properties, extending beyond isolated calculations into broader applications such as materials, catalysis, and molecular electronics. By establishing robust computational frameworks, core-electron studies continue to advance modern chemical science, paving the way for more precise experimental interpretations and the development of machine learning models for automated spectral analysis.

Accurate calculation of CEBEs for a set of small molecules is crucial not only for aiding experimental techniques such as XPS assignment but also for establishing high-quality reference data for machine learning (ML) models. Reliable theoretical CEBEs provide a systematic benchmark for interpreting experimental spectra, especially for molecules with strongly polar bonds, where standard DFT methods often introduce large errors. By generating an accurate and diverse dataset of theoretically validated CEBEs, we can train ML algorithms to predict binding energies with improved efficiency and generalization. This can lead to faster and more robust XPS assignments, reducing reliance on expensive and time-consuming ab initio calculations. In the long term, machine learning models trained on high-quality CEBE datasets will enable automated and highly accurate spectral analysis, benefiting fields such as surface science, catalysis, and materials characterization.

## Figures and Tables

**Table 1:** Comparison of key challenges of CEBE and valence ionization energy (eV) of molecules.

Factor	CEBE Challenges	Valence IPs Challenges
Electron Correlation	Stronger effects	Moderate effects
Orbital Relaxation	Critical	Less significant
Relativistic Effects	Significant (especially for heavy elements)	Small (except for heavy elements)
Charge Localization	Core-hole localization needed	Less problematic
Basis Set Dependence	Core-optimized basis required	Standard valence basis sufficient
Experimental Uncertainty	Higher (XPS calibration issues)	More precise

**Table 2:** Vertical core-electron binding energies (CEBE) of trans-1,3-pentadiene (C_5_H_8_) (in eV).

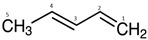	Calculated ΔDFT		Thomas et al. [110] Expt	
	Method-A *	Method-C ^#^	Adiabatic	Vertical
C1	289.743 (−0.230)	289.800 (−0.173)	289.762	289.973
C2	290.514 (−0.147)	290.571 (−0.090)	290.501	290.661
C3	290.276 (−0.136)	290.333 (−0.079)	290.247	290.412
C4	290.096 (−0.154)	290.153 (−0.097)	290.093	290.250
C5	290.727 (−0.029) + 0.057 ^c^	290.784 (+0.028)	290.565	290.756, 290.784 ^a^
RMSD	0.1533	0.1044	0.1780 ^b^	-

* Method-A (Equation (4)): with localized core hole deviation from observed correction in the brackets. ^#^ Method-C (Equation (6)): corrected CEBE ^a^ Cation − Parent + C_rel_ = 218.352863 − (−72.380309) + 0.050994 = 290.784 eV. ^b^ This RMSD indicates the discrepancies between the adiabatic and vertical ionization energies measured by experiments [110]. ^c^ Correction = 290.784 − 290.727 = +0.057 eV.

**Table 3:** Vertical carbon 1s binding energies (in eV) in small molecules *.

No	Molecule	Expt	Ref	Charge Q (C)	DFT ^a^	Δ (_DFT−Expt_)	μ/Debye ^c,d^
1	**C**H_2_=CHCH=CHCH_3_	289.97	[110]	−0.2951 ^b^	289.80 ^b^	−0.17	0.6437
2	C_6_H_5_CH_3_, C_2_, C_6_	290.11	[113]	−0.1393	290.05	−0.07	0.3688
3	C_6_H_5_CH_3_, C_4_	290.18	[113]	−0.1196	290.07	−0.11	0.3688
4	**C**H_2_=CHCH_3_	290.23	[110]	−0.3416	290.30	0.07	0.3799
5	CH_2_=CHCH=CH_2_	290.25	[110]	−0.3378	290.17	−0.07	0
6	C_6_H_5_CH_3_, C_3_, C_5_	290.25	[113]	−0.1020	290.15	−0.10	0.3688
7	CH_2_=CHCH=**C**HCH_3_	290.25	[110]	−0.1142 ^b^	290.15 ^b^	−0.10	0.6437
8	C(**C**H_3_)_4_	290.37	[114]	−0.2942 ^b^	291.36 ^b^	−0.01	0
9	H**C**CCH_3_	290.37	[115]	−0.1997	290.21	−0.16	0.7852
10	**C**_6_H_6_	290.38	[116]	−0.1519	290.35	−0.02	0
11	CH(**C**H_3_)_3_	290.39	[114]	−0.2896 ^b^	290.47 ^b^	0.08	0.1307
12	CH_2_=CH**C**H=CHCH_3_	290.41	[110]	−0.0872 ^b^	290.33 ^b^	−0.08	0.6437
13	C_6_H_5_CH_3_, C_1_	290.41	[113]	0.1041	290.35	−0.06	0.3688
14	C_6_H_5_F, C_4_	290.56	[117]	−0.1148 ^b^	290.46 ^b^	−0.09	1.4773
15	**C**H(CH_3_)_3_	290.63	[114]	−0.0141 ^b^	290.69 ^b^	0.06	0.1307
16	**C**(CH_3_)_4_	290.66	[114]	0.1231 ^b^	290.73 ^b^	0.07	0
17	CH_2_=CHCH=CHCH_3_	290.66	[110]	−0.0437 ^b^	290.00 ^h^	−0.09	0.6437
18	C_6_H_5_F, C_2_, C_6_	290.69	[117]	−0.1876 ^b^	290.60 ^b^	−0.09	1.4773
19	C_6_H_5_CH_3_, CH_3_	290.70	[113]	−0.3099	290.71	0.01	0.3688
20	**C**_2_H_6_	290.71	[118]	−0.2624	290.80	0.09	0
21	C_6_H_5_F, C_3_, C_5_	290.75	[117]	−0.0981	290.66	−0.10	1.4773
22	CH_2_=CHCH=CHCH_3_	290.76	[110]	−0.2931 ^b^	290.78 ^b^	0.03	0.6437
23	CH_2_=**C**HCH_3_	290.76	[110]	−0.1331	290.80	0.04	0.3799
24	**C**_2_H_4_	290.82	[118]	−0.2838	290.94	0.12	0
25	**C**H_4_	290.84	[118]	−0.3664	290.96	0.11	0
26	CH_2_=CHCH=CH_2_	290.85	[110]	−0.0900	290.85	0.01	0
27	CH_2_=CH**C**H_3_	290.86	[110]	−0.2617	290.93	0.06	0.3799
28	HCCCH_3_	290.93	[115]	−0.0736	290.91	−0.02	0.7852
29	**C**H_2_=CHCl	290.93	[119]	−0.2973	290.85	−0.08	1.6863
30	**C**H_3_CHFCH_3_	290.97	[114]	−0.2895	290.95	−0.02	1.8801
31	CH_2_=CCl_2_	290.99	[119]	−0.2795	290.81	−0.18	1.6013
32	CH_3_CH_2_F	291.13	[114]	−0.2713	291.26	0.14	2.0770
33	C_2_H_2_	291.25	[118]	−0.2008	291.48	0.24	0
34	HCC**C**H_3_	291.76	[115]	−0.2725	291.85	0.09	0.7852
35	H**C**CCF_3_	292.03	[115]	−0.1213	292.05	0.03	2.5410
36	**C**H_3_Br	292.06	[120]	−0.1950	292.13	0.07	2.1523
37	HC**C**CF_3_	292.14	[115]	−0.1954	292.17	0.02	2.5410
38	cis CHCl=CHCl	292.27	[119]	−0.0873	292.21	−0.06	2.1910
39	**C**HCl=CCl_2_	292.29	[119]	−0.0646	292.11	−0.18	2.0770
40	CH_2_=**C**HCl	292.29	[119]	−0.0590	292.28	−0.01	1.0352
41	Trans CHCl=CHCl	292.34	[119]	−0.0741	292.19	−0.15	1.6863
42	**C**H_3_Cl	292.43	[114]	−0.1439	292.52	0.09	0
43	C_6_H_5_F, C_1_	292.81	[117]	0.2941	292.66	−0.15	2.1375
44	CH_2_Br_2_	293.10	[120]	0.0856	293.11	0.01	1.4773
45	CH_3_**C**HFCH_3_	293.15	[114]	0.2113 ^b^	293.02 ^b^	−0.13	1.7209
46	CH_3_**C**H_2_F	293.28	[114]	0.1915	293.28	−0.01	1.8801
47	**C**H_2_ClBr	293.46	[120]	−0.0343	293.49	0.03	2.0770
48	**C**_2_Cl_4_	293.51	[119]	0.1178	293.32	−0.19	1.8105
49	CHCl=**C**Cl_2_	293.53	[119]	0.0987	293.38	−0.15	1.0352
50	**C**H_3_F	293.56	[118]	0.1151	293.57	0.01	2.0326
51	CH_2_=**C**Cl_2_	293.56	[119]	0.1249	293.52	−0.04	1.6013
52	**C**H_2_Cl_2_	293.86	[114]	0.0173	**293.89**	**0.03**	1.8889
53	CHBr_3_	293.99	[120]	−0.0371	294.00	0.0	1.0487
54	**C**H_2_FCl	295.04	[120]	0.2545	294.98	−0.06	2.0708
55	**C**HCl_3_	295.14	[114]	0.1226	295.13	0.02	1.2548
56	**C**Cl_3_Br	295.91	^G^	0.1343	295.90	−0.01	0.1678
57	**C**O	296.23	[118]	0.0856	296.31	0.09	0.2838
58	**C**FBr_3_	296.28	[120]	0.2643	296.18	−0.10	0.6251
59	**C**Cl_4_	296.32	[114]	0.1991	296.28	−0.03	0
*60*	***C*** *H*_2_ *F*_2_	*296.35*	[120]	*0.4331*	*296.16*	*−0.19*	*2.1557*
*61*	***C*** *HFCl*_2_	*296.37*	[120]	*0.3371*	*296.23*	*−0.14*	*1.4649*
*62*	***C*** *O*_2_	*297.70*	[118]	*0.5394*	*297.29*	*−0.41*	*0*
*63*	***C*** *HF*_2_ *Cl*	*297.70*	[120]	*0.4977*	*297.43*	*−0.27*	*1.6383*
*64*	***C*** *F*_2_ *Br*_2_	*297.87*	[114]	*0.3103*	*297.67*	*−0.20*	*0.6795*
*65*	***C*** *HF*_3_	*299.16*	[118]	*0.6132*	*298.72*	*−0.44*	*1.8030*
*66*	*HCC**C**F_3_*	*299.55*	[115]	*0.7613*	*299.01*	*−0.54*	*2.5410*
*67*	***C*** *F*_3_ *Br*	*299.74*	[114]	*0.6398*	*299.29*	*−0.45*	*0.6395*
*68*	***C*** *F*_4_	*301.90*	[118]	*0.7352*	*301.19*	*−0.71*	*0*

* Note that reliable CEBEs for N1s, O1s, and F1s from synchrotron experiments are not available. RMSD = 0.1735 eV. It is noted that Kahk et al. calculated 22 CEBEs previously [30]. The bold carbons atoms in the table indicated the target carbon atoms and the italic at the bottom of the table indicates that they are halogenated molecules. ^a^ ∆DFTpw/et-pVQZ//CCSD(T)/cc-pVTZ + C_rel_. ^b^ ∆DFTpw/et-pVQZ//B3LYP/cc-pVTZ + C_rel_ ^G^ Borve, K.J., private communication (June 2021) [121]. ^c,d^ Calculated dipole moment using HF/cc-pVTZ and the highlighted with blue color are obtained using B3LYP/cc-pVTZ. Note that the wavefunction of the post-HF method CCSD(T) is the HF wavefunction so the dipole moments are calculated using the HF wavefunction.

**Table 4:** Comparison of the performance on C1s CEBE of DFTpw, mPW1P, and PBE50 DFT functionals with more exchange energy (eV).

Case	Molecule	PW86-PW91 (DFTpw)	Abs	PBE50	Abs	mPW1PW	Abs
60	**C**H_2_F_2_	−0.188	0.188	−0.140	0.140	−0.125	0.125
61	**C**HFCl_2_	−0.143	0.143	0.076	0.076	0.020	0.020
62	**C**O_2_	−0.407	0.407	0.475	0.475	0.104	0.104
63	**C**HF_2_Cl	−0.278	0.278	−0.007	0.007	−0.085	0.085
64	**C**F_2_Br_2_	−0.200	0.200	0.247	0.247	0.115	0.115
65	**C**HF_3_	−0.439	0.439	−0.093	0.093	−0.201	0.201
66	HCC**C**F_3_	−0.538	0.538	0.024	0.024	−0.171	0.171
67	**C**F_3_Br	−0.446	0.446	0.084	0.084	−0.093	0.093
68	**C**F_4_	−0.710	0.710	−0.042	0.042	−0.286	0.286
Sum			3.349		1.188		1.200
AAD			0.372		0.132		0.133

The bold carbons atoms in the table indicated the target carbon atoms.

## Data Availability

The original contributions presented in this study are included in the article/Supplementary Material.

## Supplementary Materials

The following supporting information can be downloaded at: https://www.mdpi.com/article/10.3390/molecules30132887/s1, Table S1: Optimized Cartesian coordinates of molecules; Figure S1: The excess individual C1s CEBEs of the molecules with respect to their corresponding measured C1s energies. Most of the excess errors are within the CEBE ≤ ±0.1 eV box (RMSD = 0.1735 eV); Figure S2: Comparison of C1s CEBEs of small hydrocarbons with respect to their corresponding measured C1s energies (eV) (RMSD = 0.0946 eV); Figure S3: Comparison of C1s CEBEs of the small halogenated hydrocarbons with respect to their corresponding measured C1s energies (eV) (RMSD = 0.2143 eV).
