# Supplementary material for: The Role of Exchange Energy in Modeling Core-Electron Binding Energies of Strongly Polar Bonds
Source: Molecules. 2025 Jul 7;30(13):2887. doi: 10.3390/molecules30132887 (PMC12251301; doi:10.3390/molecules30132887)
Supplement: Supplementary file 1 [file molecules-30-02887-s001.zip › molecules-3706730-supplementary.pdf]

The Role of Exchange Energy in Modeling Core-Electron Binding Energies of Strongly Polar Bonds

Feng Wang[1] and Delano P. Chong[2]*

[1]School of Science, Computing and Emerging Technologies, Swinburne University of Technology, Melbourne, Victoria 3122, Australia

[2]Department of Chemistry, University of British Columbia, 2016 Main Mall, Vancouver, B.C. V6T 1Z1, Canada

*Corresponding author: fwang@swin.edu.au

Supplementary Materials

Table S1 Optimized Cartesian coordinates of molecules

CH2=CH-CH=CH2-CH3//B3LYP/cc-pVTZ

```
6 -2.457674 -0.310623 0.000000
6 -0.992311 -0.648356 0.000000
6 0.000000 0.246783 0.000000
6 1.409306 -0.096896 0.000000
6 2.408244 0.788389 0.000000
1 2.217926 1.854762 0.000000
1 3.443017 0.476083 0.000000
1 1.642825 -1.157773 0.000000
1 -0.238519 1.307853 0.000000
1 -0.730230 -1.702055 0.000000
1 -3.073492 -1.208649 0.000000
1 -2.733455 0.276998 0.878649
1 -2.733455 0.276998 -0.878649
```

Propene//CCSD(T)/cc-pVTZ

```
6 1.143393 -0.502472 0.000000
6 0.000000 0.486444 0.000000
6 -1.293239 0.141692 0.000000
1 -1.601252 -0.898844 0.000000
1 -2.075107 0.890535 0.000000
1 0.255134 1.541608 0.000000
1 2.107980 0.005338 0.000000
1 1.106160 -1.146312 0.881560
1 1.106160 -1.146312 -0.881560
```

Butadiene//CCSD(T)/cc-pVTZ

```
6 0.599689 1.743615 0.000000
6 0.599689 0.409819 0.000000
```

```
6 -0.599689 -0.409819 0.000000
6 -0.599689 -1.743615 0.000000
1 -1.519591 -2.311187 0.000000
1 0.324666 -2.308263 0.000000
1 -1.546169 0.122340 0.000000
1 1.546169 -0.122340 0.000000
1 -0.324666 2.308263 0.000000
1 1.519591 2.311187 0.000000
```

C(CH3)4//B3LYP/cc-pVTZ

```
6 0.000000 0.000000 0.000000
6 0.886772 0.886772 0.886772
1 1.529876 1.529876 0.282378
1 0.282378 1.529876 1.529876
1 1.529876 0.282378 1.529876
6 -0.886772 -0.886772 0.886772
1 -1.529876 -1.529876 0.282378
1 -0.282378 -1.529876 1.529876
1 -1.529876 -0.282378 1.529876
6 0.886772 -0.886772 -0.886772
1 0.282378 -1.529876 -1.529876
1 1.529876 -0.282378 -1.529876
1 1.529876 -1.529876 -0.282378
6 -0.886772 0.886772 -0.886772
1 -1.529876 0.282378 -1.529876
1 -1.529876 1.529876 -0.282378
1 -0.282378 1.529876 -1.529876
```

Propyne//CCSD(T)/cc-pVTZ

```
6 0.000000 0.000000 1.429756
6 0.000000 0.000000 0.218608
6 0.000000 0.000000 -1.247915
1 0.000000 1.021188 -1.631692
1 0.884375 -0.510594 -1.631692
1 -0.884375 -0.510594 -1.631692
1 0.000000 0.000000 2.492387
```

Benzene//CCSD(T)/cc-pVTZ

```
6 0.000000 1.397546 0.000000
6 1.210310 0.698773 0.000000
6 1.210310 -0.698773 0.000000
6 0.000000 -1.397546 0.000000
6 -1.210310 -0.698773 0.000000
6 -1.210310 0.698773 0.000000
1 -2.148327 1.240337 0.000000
1 -2.148327 -1.240337 0.000000
1 0.000000 -2.480674 0.000000
1 2.148327 -1.240337 0.000000
1 2.148327 1.240337 0.000000
1 0.000000 2.480674 0.000000
```

CH(CH3)3 //B3LYP/cc-pVTZ

```
6 0.000000 0.000000 0.371313
```

```
6 0.000000 1.458183 -0.095475
1 0.882092 1.990957 0.264254
1 0.000000 1.516159 -1.187152
1 -0.882092 1.990957 0.264254
6 -1.262824 -0.729092 -0.095475
1 -1.283173 -1.759393 0.264254
1 -2.165265 -0.231564 0.264254
1 -1.313032 -0.758080 -1.187152
6 1.262824 -0.729092 -0.095475
1 1.283173 -1.759393 0.264254
1 1.313032 -0.758080 -1.187152
1 2.165265 -0.231564 0.264254
1 0.000000 0.000000 1.466614
```

C6H5F//B3LYP/cc-pVTZ

```
6 0.000000 1.203399 -1.131480
6 0.000000 1.212403 0.258960
6 0.000000 0.000000 0.925724
6 0.000000 -1.212403 0.258960
6 0.000000 -1.203399 -1.131480
6 0.000000 0.000000 -1.828464
1 0.000000 0.000000 -2.909537
1 0.000000 -2.141731 -1.669311
1 0.000000 -2.133621 0.823647
9 0.000000 0.000000 2.276394
1 0.000000 2.133621 0.823647
1 0.000000 2.141731 -1.6693
```

Ethane//CCSD(T)/cc-pVTZ

```
6 0.000000 0.000000 0.764514
6 0.000000 0.000000 -0.764514
1 0.000000 -1.017962 -1.159455
1 -0.881581 0.508981 -1.159455
1 0.881581 0.508981 -1.159455
1 0.000000 1.017962 1.159455
1 -0.881581 -0.508981 1.159455
1 0.881581 -0.508981 1.159455
```

CH4//CCSD(T)/cc-pVTZ

```
6 0.000000 0.000000 0.000000
1 0.628118 0.628118 0.628118
1 -0.628118 -0.628118 0.628118
1 0.628118 -0.628118 -0.628118
1 -0.628118 0.628118 -0.628118
```

CH2=CHCl//CCSD(T)/cc-pVTZ

```
6 1.305736 1.029255 0.000000
6 0.000000 0.762449 0.000000
17 -0.632389 -0.857487 0.000000
1 -0.768896 1.521471 0.000000
1 2.050331 0.245547 0.000000
1 1.634759 2.060042 0.000000
```

CH3-CHF-CH3//B3LYP/cc-pVTZ

```
6 -0.280726 -0.583860 1.272187
6 -0.280726 0.238511 0.000000
6 -0.280726 -0.583860 -1.272187
1 -0.230767 0.065190 -2.145811
1 0.576548 -1.257742 -1.289793
1 -1.191726 -1.180776 -1.338830
9 0.874566 1.043321 0.000000
1 -1.126146 0.932016 0.000000
1 -1.191726 -1.180776 1.338830
1 0.576548 -1.257742 1.289793
1 -0.230767 0.065190 2.14581
```

CH2=CCl2//CCSD(T)/cc-pVTZ

```
6 0.000000 0.000000 0.418334
6 0.000000 0.000000 1.751698
1 0.000000 0.934803 2.293237
1 0.000000 -0.934803 2.293237
17 0.000000 1.458450 -0.517843
17 0.000000 -1.458450 -0.517843
```

CCl2=CHCl//CCSD(T)/cc-pVTZ

```
6 -1.042614 -0.397553 0.000000
6 0.000000 0.442073 0.000000
17 -0.270365 2.152594 0.000000
17 1.645597 -0.055895 0.000000
17 -0.886495 -2.111291 0.000000
1 -2.052843 -0.019047 0.000000
```

CH3CH2F//CCSD(T)/cc-pVTZ

```
6 1.122503 -0.455323 0.000000
6 0.000000 0.558323 0.000000
9 -1.224654 -0.098259 0.000000
1 0.034630 1.192755 0.888147
1 0.034630 1.192755 -0.888147
1 2.087047 0.057148 0.000000
1 1.065281 -1.088162 0.886255
1 1.065281 -1.088162 -0.886255
```

Ethyne//CCSD(T)/cc-pVTZ

```
6 0.000000 0.000000 0.601602
6 0.000000 0.000000 -0.601602
1 0.000000 0.000000 -1.667783
1 0.000000 0.000000 1.667783
```

HCCCF3//CCSD(T)/cc-pVTZ

```
6 0.000000 0.000000 2.337656
6 0.000000 0.000000 1.130051
```

```
6 0.000000 0.000000 -0.340341
9 0.000000 1.246269 -0.820948
9 1.079300 -0.623134 -0.820948
9 -1.079300 -0.623134 -0.820948
1 0.000000 0.000000 3.401402
```

CH3Br//cc-pVTZ

```
6 0.000000 0.000000 -1.521145
35 0.000000 0.000000 0.419907
1 0.000000 1.032019 -1.856630
1 0.893754 -0.516009 -1.856630
1 -0.893754 -0.516009 -1.856630
```

cis CHCl=CHCl//CCSD(T)/cc-pVTZ

```
6 0.000000 0.667570 0.965737
6 0.000000 -0.667570 0.965737
17 0.000000 -1.647877 -0.452478
1 0.000000 -1.213380 1.897697
17 0.000000 1.647877 -0.452478
1 0.000000 1.213380 1.897697
```

trans CHCl=CHCl//CCSD(T)/cc-pVZ

```
6 -0.368797 0.555165 0.000000
6 0.368797 -0.555165 0.000000
17 -0.368797 -2.123307 0.000000
1 1.448557 -0.554557 0.000000
17 0.368797 2.123307 0.000000
1 -1.448557 0.554557 0.000000
```

CH3Cl//CCSD(T)/cc-pVTZ

```
6 0.000000 0.000000 -1.131309
17 0.000000 0.000000 0.659506
1 0.000000 1.030298 -1.474580
1 0.892264 -0.515149 -1.474580
1 -0.892264 -0.515149 -1.474580
```

CH2=CBr2//CCSD(T)/cc-pVTZ

```
6 0.000000 0.000000 0.933457
35 0.000000 -1.613968 -0.124009
35 0.000000 1.613968 -0.124009
1 -0.897100 0.000000 1.539941
1 0.897100 0.000000 1.539941
```

CH3F//CCSD(T)/cc-pVTZ

```
6 0.000000 0.000000 -0.630997
9 0.000000 0.000000 0.750176
1 0.000000 1.029917 -0.988532
1 0.891934 -0.514959 -0.988532
```

1 -0.891934 -0.514959 -0.988532

CH2Cl2//CCSD(T)/cc-pVTZ

6 0.000000 0.000000 0.769974
17 0.000000 1.478187 -0.216938
17 0.000000 -1.478187 -0.216938
1 0.896734 0.000000 1.378018
1 -0.896734 0.000000 1.378018

CHBr3//CCSD(T)/cc-pVTZ
6 0.000000 0.000000 0.523814
1 0.000000 0.000000 1.604945
35 0.000000 1.843927 -0.045217
35 1.596887 -0.921963 -0.045217
35 -1.596887 -0.921963 -0.045217

CH2FCl//CCSD(T)/cc-pVTZ

6 0.000000 0.806388 0.000000
9 1.358346 0.756201 0.000000
17 -0.678826 -0.838392 0.000000
1 -0.342535 1.304268 0.902665
1 -0.342535 1.304268 -0.902665

C2Cl4//CCSD(T)/cc-pVTZ

6 0.494422 0.446856 0.000018
6 -0.494439 -0.446850 -0.000013
17 -2.154646 0.049996 -0.000004
1 -0.333877 -1.514798 0.000104
17 2.154653 -0.049999 -0.000006
1 0.333860 1.514807 0.000049

CHCl3//CCSD(T)/cc-pVTZ

6 0.000000 0.000000 -0.458564
17 0.000000 -1.687699 0.084150
17 -1.461590 0.843849 0.084150
17 1.461590 0.843849 0.084150
1 0.000000 0.000000 -1.540275

CCl3Br//C CSD(T)/cc-pVTZ

6 0.000000 0.000000 -0.411426
35 0.000000 0.000000 1.532854
17 0.000000 1.674666 -1.003556
17 1.450303 -0.837333 -1.003556
17 -1.450303 -0.837333 -1.003556

CO//CCSD(T)/cc-pVTZ

6 0.000000 0.000000 -0.648997
8 0.000000 0.000000 0.486748

CFBr3//CCSD(T)/cc-pVTZ

```
6 0.000000 0.000000 0.429997
9 0.000000 0.000000 1.771392
35 0.000000 1.838803 -0.176405
35 1.592450 -0.919402 -0.176405
35 -1.592450 -0.919402 -0.176405
```

CCl4//CCSD(T)/cc-pVTZ

```
6 0.000000 0.000000 0.000000
17 1.025873 1.025873 1.025873
17 -1.025873 -1.025873 1.025873
17 1.025873 -1.025873 -1.025873
17 -1.025873 1.025873 -1.025873
```

CH2F2//CCSD(T)/cc-pVTZ

```
6 0.000000 0.000000 -0.501130
9 0.000000 1.100500 0.289538
9 0.000000 -1.100500 0.289538
1 -0.908616 0.000000 -1.102452
1 0.908616 0.000000 -1.102452
```

CHFCl2//CCSD(T)/cc-pVTZ

```
6 0.177653 0.523304 0.000000
9 -0.909022 1.316528 0.000000
1 1.075054 1.131314 0.000000
17 0.177653 -0.474114 1.463132
17 0.177653 -0.474114 -1.463132
```

CO2//CCSD(T)/cc-pVTZ

```
6 0.000000 0.000000 0.000000
8 0.000000 0.000000 1.166300
8 0.000000 0.000000 -1.166300
```

CHF2Cl//CCSD(T)/cc-pVTZ

```
6 -0.565059 -0.092416 0.000000
9 -0.565059 -0.875761 1.084574
9 -0.565059 -0.875761 -1.084574
17 0.882819 0.928013 0.000000
1 -1.446503 0.541975 0.00000
```

CHF3//CCSD(T)/cc-pVTZ

```
6 0.000000 0.000000 -0.336645
9 0.000000 -1.249946 0.127539
9 -1.082485 0.624973 0.127539
9 1.082485 0.624973 0.127539
1 0.000000 0.000000 -1.423692
```

CF3Br//CCSD(T)/cc-pVTZ

```
6 0.000000 0.000000 -0.811165
35 0.000000 0.000000 1.117997
9 0.000000 1.243131 -1.268996
9 1.076583 -0.621566 -1.268996
9 -1.076583 -0.621566 -1.268996
```

CF4//CCSD(T)/cc-pVTZ

```
6 0.000000 0.000000 0.000000
9 0.761637 0.761637 0.761637
9 -0.761637 -0.761637 0.761637
9 0.761637 -0.761637 -0.761637
9 -0.761637 0.761637 -0.761637
```

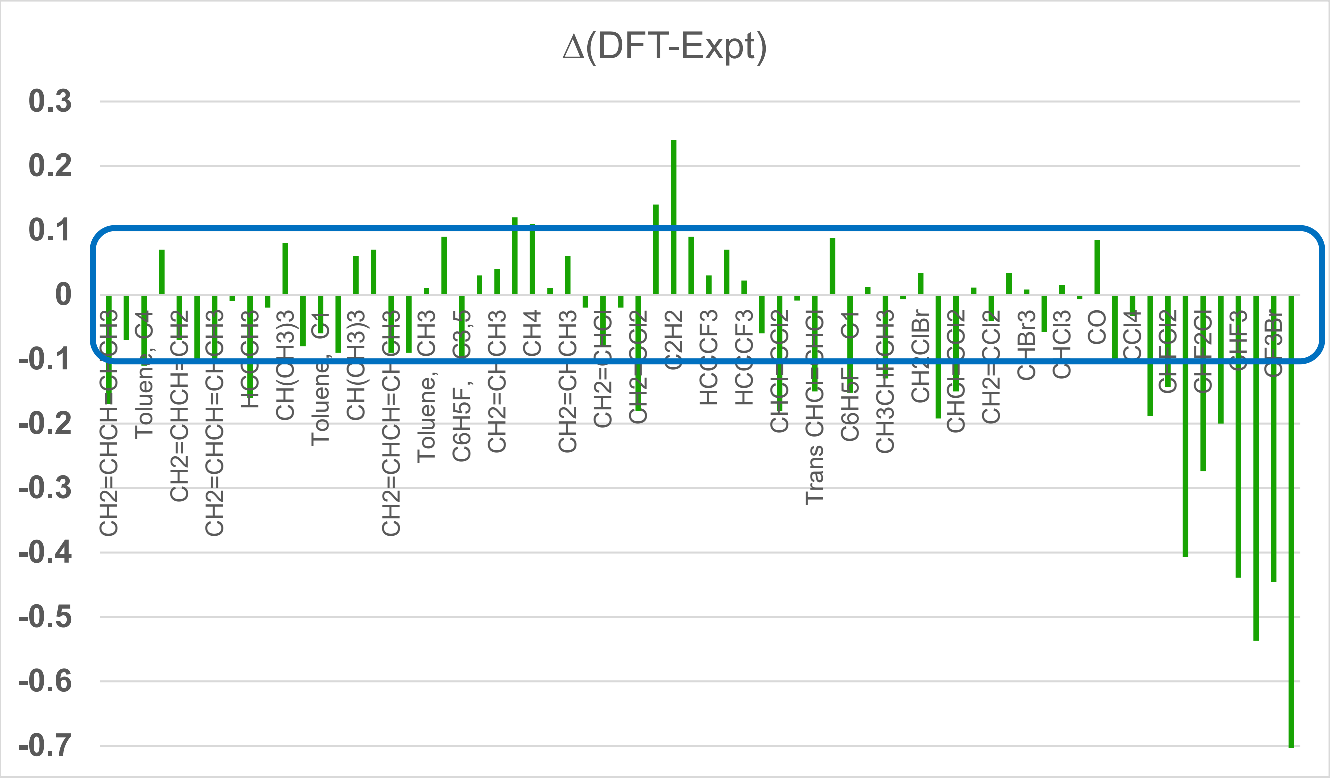

Figure S1 The excess individual C1s CEBEs of the molecules with respect to their corresponding measured C1s energies. Most of the excess errors are within the CEBE ≤± 0.1 eV box (RMSD = 0.1735 eV).

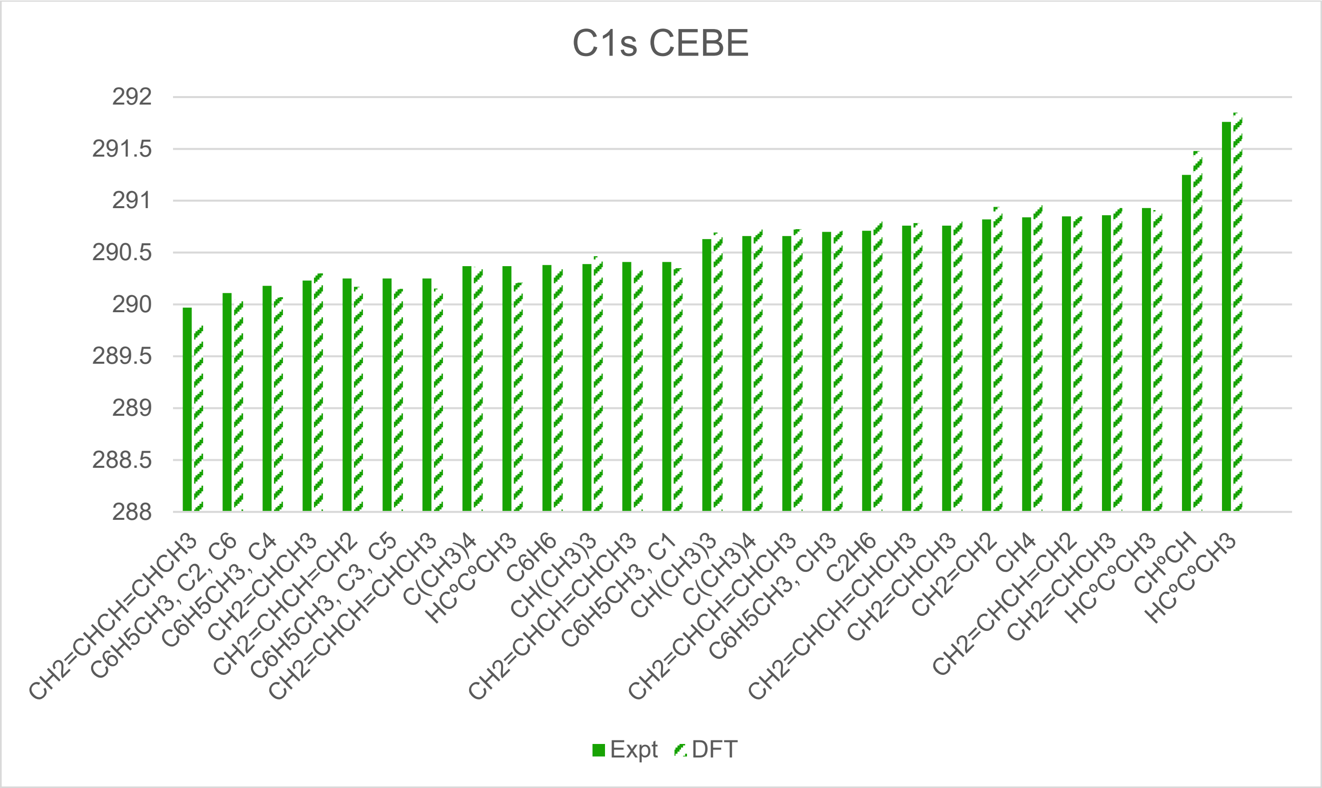

Figure S2 Comparison of C1s CEBEs of small hydrocarbons with respect to their corresponding measured C1s energies (eV) (RMSD = 0.0946 eV).

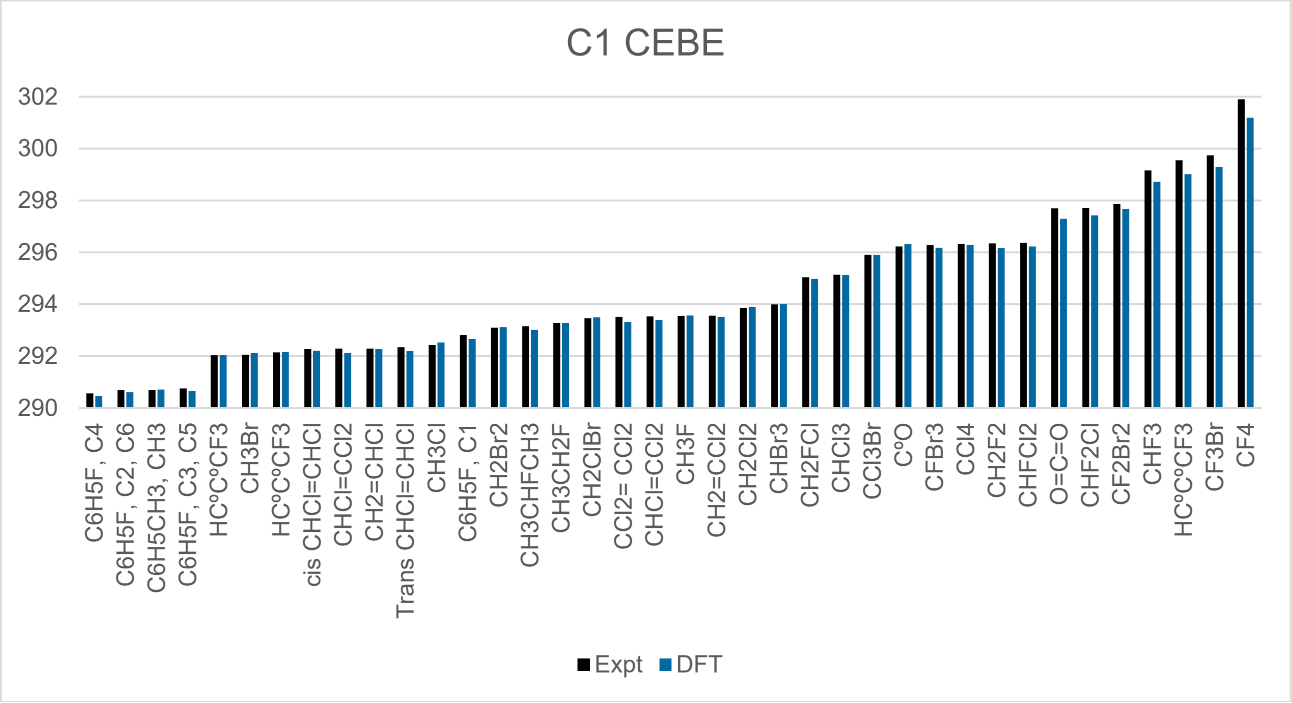

Figure S3 Comparison of C1s CEBEs of the small halogenated hydrocarbons with respect to their corresponding measured C1s energies (eV) (RMSD = 0.2143 eV).
